# Delta-9 fatty acid desaturase mutants display increased body size

**DOI:** 10.17912/SS8E-6587

**Published:** 2018-09-18

**Authors:** James F. Clark, Cathy Savage-Dunn

**Affiliations:** 1 Department of Biology, Queens College, CUNY 65-30 Kissena Boulevard Flushing, NY 11367; 2 Ph.D. Program in Biology, The Graduate Center, CUNY 365 5th Avenue New York, NY 10016

**Figure 1.**
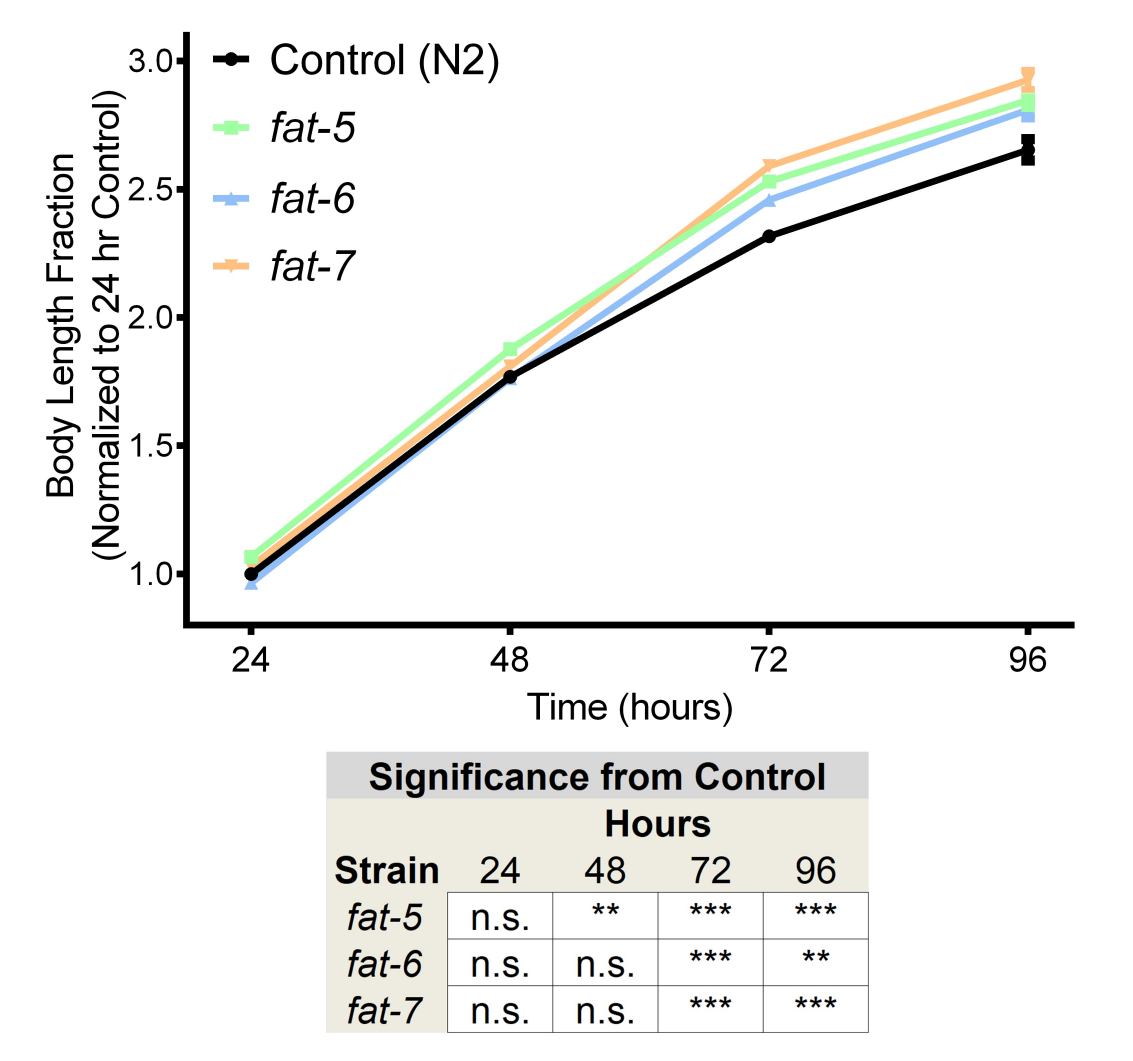


## Description

*C. elegans* contain three Δ9 fatty acid desaturases (FADs), *fat-5*, *fat-6*, and *fat-7*, responsible for the initial desaturation step in the synthesis of complex polyunsaturated fatty acids (PUFAs). Production of PUFAs is vital for survival, as simultaneous loss-of-function in all three genes results in synthetic lethality (Brock et al. 2006). Animals containing mutations in any one of the three Δ9 FADs, *fat-5*, *fat-6*, and *fat-7*, display an increase in body length. *fat-5* mutants display a significant increase from 48 hours onward, while *fat-6* and *fat-7* display a significant increase from 72 hours onward. Since *fat-6* and *fat-7* mutants have reduced lipid accumulation (Horikawa et al. 2008; Zhang et al. 2013; Clark et al. 2018), these findings on body size suggest that the two phenotypes are not correlated. Consistent with this conclusion, *dbl-1* mutants display both decreased body size and lipid accumulation while *lon-2* and *dbl-1(OE)* mutants display increased body size and decreased lipid accumulation at the L4 stage (Clark et al. 2018). Error bars denote SEM, Statistical significance determined via Student’s T-test, n.s. not significant, * p<0.05, ** p<.0.01,*** p<0.001. n>25 per strain, repeated in duplicate. Animals were synchronized via an egg lay followed by a timed hatch. Animals were collected and imaged at 25X using a QImaging Retiga EXi camera with QCapture software at each timepoint. Body length was measured by tracing the midline of the animals in ImageJ.

## Reagents

Strains: N2. Alleles: *fat-5 (tm420), fat-6 (tm331)*, *fat-7 (wa36)*.
